# Paralysis Treated by Nerve Stretching

**Published:** 1877-03

**Authors:** 


### PARALYSIS TREATED BY NERVE STRETCHING.

The *Aerztliches Intelligenz-Blatt*, No. 8, 1876, reports the following case:—The patient, a Polish gentleman, aged thirty-five, had for eleven years suffered from paraplegia, the result of an injury in the sacral region. There had been an almost total loss of sensation, while voluntary motion was completely annihilated. The bladder and rectum were affected, and incontinence of the urine followed. After administering chloroform, the following operation was performed: A curved incision was made in the right groin, over and along the course of Poupart's ligament. The fascia was divided, and the anterior crural nerve exposed and separated from the vein and artery. The operator hooked his finger under the nerve, and raised it with such force that the foot was moved. He then seized it between the thumb and finger, and made traction downward, until it appeared to be elongated. The inguinal wound having been carefully dressed, a longitudinal incision was made on the same side, midway between the tuberischii and the great trochanter, so as to expose the sciatic nerve, which was also elevated from its bed and pulled forcibly upward and downward. These operations were followed by the immediate cessation of the spasmodic movements with which the limbs had been affected since the time of the accident, on the side on which the operation had been performed. The wounds healed rapidly, and the operation was repeated on the left side in a fortnight, with the most satisfactory result. The relief afforded was complete, and the patient who for years had been confined to his bed, was subsequently able to get up and move about on crutches, the paralyzed limbs being furnished with mechanical support.—*Med. & Surg. Reporter.*

